# Metal–Polymer Nanocomposites: A Promising Approach to Antibacterial Materials

**DOI:** 10.3390/polym15092167

**Published:** 2023-05-02

**Authors:** Asma Ghazzy, Rajashri R. Naik, Ashok K. Shakya

**Affiliations:** 1 Faculty of Pharmacy, Al-Ahliyya Amman University, Amman 19328, Jordan; a.alghazzy@ammanu.edu.jo; 2 Pharmacological and Diagnostic Research Center, Faculty of Pharmacy and Allied Medical Sciences, Al-Ahliyya Amman University, Amman 19328, Jordan; rsharry@ammanu.edu.jo; 3 Faculty of Allied Medical Sciences, Al-Ahliyya Amman University, Amman 19328, Jordan

**Keywords:** metal oxides, metal–polymer nanocomposites, silver, zinc, copper and gold, nanoparticles, electrospinning, chemical reduction, sol–gel, antibacterial, biomedical application

## Abstract

There has been a new approach in the development of antibacterials in order to enhance the antibacterial potential. The nanoparticles are tagged on to the surface of other metals or metal oxides and polymers to achieve nanocomposites. These have shown significant antibacterial properties when compared to nanoparticles. In this article we explore the antibacterial potentials of metal-based and metal–polymer-based nanocomposites, various techniques which are involved in the synthesis of the metal–polymer, nanocomposites, mechanisms of action, and their advantages, disadvantages, and applications.

## 1. Introduction

Antibiotics are designed to prevent bacterial infection, depending on the four mechanisms or the activities that the bacteria carry out. However, there are some limitations, such as difficulty in penetrating into the bacterial cell and excreting of these antibiotics from the system. To overcome these limitations, there is a new approach in this regard; new antibacterials with nanoparticles have been developed based on the existing mechanism of the antibacterials and these antibacterials loaded with nanoparticles have shown significant antibacterial potencies [1]. In recent years, there has been a new approach developed in this regard to enhance the antibacterial potency by tagging the nanoparticles on the surface of other metal oxides or polymers to acquire nanocomposites. The literature survey and the research on these nanocomposites show that nanocomposites exhibit more efficient antibacterial properties when compared to nanoparticles alone. Composite materials combine the favorable properties of a matrix and filler, and can lead to new functionalities, especially due to the size of the filers which are in nanoscale. These composite materials are now popularly known as nanocomposites. One of the approaches towards synthesizing the nanocomposites is to tag the nanoparticles with either metal oxide or polymers. Polymers are one of the choices; polymers are a popular choice as the matrix component due to their low cost, versatility, and ability to be easily processed into various shapes, including thin films. While much research has been carried out on structural polymer-based nanocomposites, there have been fewer investigations on polymer nanocomposites for functional applications. Of particular interest are nanocomposites that contain metal nanoparticles dispersed in a dielectric matrix, which offer novel properties and have various new applications such as electronic conductivity, optical features, magnetic properties, and enhanced catalytic activity. 

**Figure 1:**
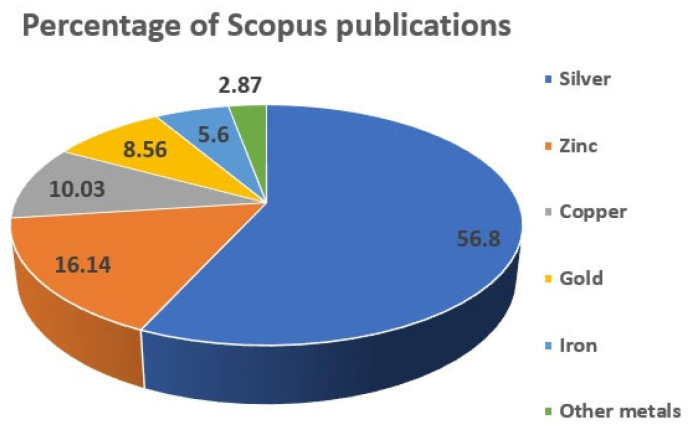
Percentage of antibacterial studies of metal nanoparticles. **Source**: Scopus Database. Keywords: metal (silver, zinc, copper, gold, iron, and other metals), nanoparticles, antibacterial.

It may be noted that the investigation of antibacterial properties of metal nanoparticles started with silver and copper in 2003, followed by gold nanoparticles in the same year. The number of Scopus publications related to metal nanoparticles research has increased significantly since then, with silver being the most studied metal and having the highest percentage of Scopus publications at 56.8%. Zinc, copper, and gold were also investigated, but to a lesser extent, with 16.1%, 10%, and 8.6% of the Scopus publications, respectively (Figure 1). Iron and other metals were also studied but had lower percentages of Scopus publications. The data suggest that the scientific community has shown significant interest in investigating the antibacterial properties of metal nanoparticles. Silver has emerged as the most extensively studied metal nanoparticle for antibacterial purposes, possibly due to its broad-spectrum antimicrobial activity, low toxicity, and relatively lower cost than gold. The lower number of publications for other metals may suggest that they are less promising compared to silver, but it is essential to note that this may not reflect the quality or significance of the research. 

This review highlights the potential of metal nanoparticles as promising antibacterial agents. Metal-based nanocomposites’ bacterial action, their mechanism of action, various mechanisms that are currently employed in the synthesis of the nanocomposites, potential of metal–polymer-based nanocomposites, their challenges and their application in various fields are investigated, and this sets the groundwork for further research in this field. We conclude the article with a positive approach in developing therapeutically enhanced antibacterials.

## 2. Antibacterial Mechanism of Action of Metal–Polymer Nanocomposites

Antibacterial mechanisms of metal nanoparticles and metal oxide nanoparticles nanocomposites are discussed in this section. Among metals, silver, copper, zinc, and gold nanocomposites are discussed here in this section. We also briefly explain the antiviral property of some of the NPs.

**Figure 2:**
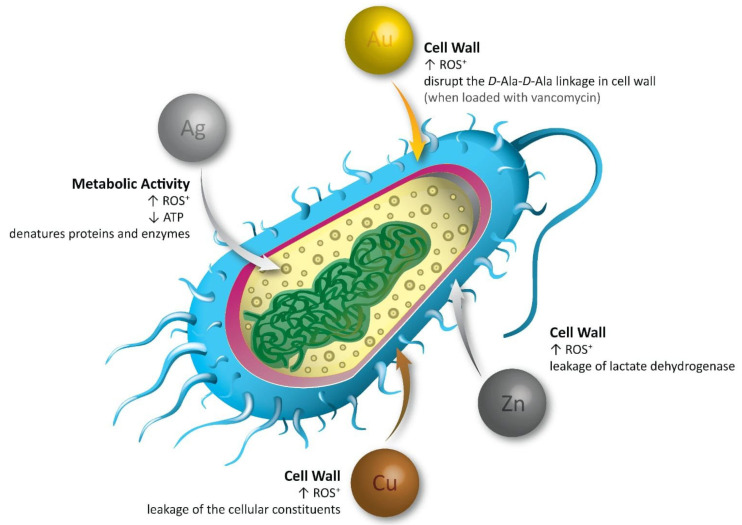
Mechanisms of action of silver, gold, zinc, and copper nanoparticles as antibacterial agents.

Gold and silver nanoparticles have been shown to possess significant amounts of antibacterial activity when compared to other metal nanoparticles. The antibacterial activity of the nanocomposite depends on the interaction between the chemical released by the nanocomposite and between the bacteria. Figure 2 shows the different mechanisms of action of metallic nanoparticles as antibacterials. 

### 2.1. Antibacterial Mechanism of Action of Silver Nanocomposites 

The silver nanoparticle exhibits antibacterial activity through various mechanisms such as interfering with the metabolic activity, enzymes, denaturation of protein, condensation of DNA, decrease in ATP production, production of ROS, and causing oxidative stress. Bao et al. [2] showed that silver-based graphene oxide nanocomposite sheets showed high rates of antibacterial activity. They attributed this to the release of silver nanoparticles from the graphene nanocomposite sheets that caused the destruction of the protein and the DNA of the bacteria, resulting in death of the bacteria. In a similar study involving silver graphene oxide nanocomposite sheets, it showed bactericidal properties. In another study, the antibacterial activity of graphene-oxide-based silver nanocomposites and graphene oxide decorated with L-cysteine nanocomposites exhibited activity through different mechanisms against Gram-positive and Gram-negative bacteria. It had bacteriostatic effect against Gram-positive bacteria, and Gram-negative bacteria died of cell wall destruction. This difference in the mechanism was attributed to the difference in the cell wall component and the structure [3]. The cysteine and silver nanocomposites interfere with the metabolic processes in Gram-positive bacteria by interacting with the thiol group of the enzyme, and fragmentation of DNA was caused by interacting with phosphorus in the nucleic acid. In Gram-negative, it caused damage to the cell wall by causing oxidative and mechanical stress to the cell wall [4]. Considering copper nanoparticles, it produces destruction in the helical structured links of DNA within and in between its strands.

### 2.2. Antibacterial Mechanism of Action of Copper Nanocomposites 

Copper nanoparticles are shown to possess the antibacterial properties due to the release of monovalent and divalent copper ions from the nanoparticles that damage the cell wall of the bacteria. Usually, metals such as gold and silver are tagged to enhance the antibacterial properties, but as gold and silver are costlier than other metals, copper would be an excellent choice in the synthesis of nanocomposites. Carboxymethyl cellulose coupled with copper oxide nanocomposites exhibited an appreciable amount of antibacterial activity against both Gram-positive and Gram-negative bacteria. The mechanism by which it caused the antibacterial activity caused the leakage of the cellular constituents that killed the bacteria [5]. The antibacterial mechanism of zeolite nanocomposite along with zinc oxide and copper oxide was due to the release of the ions from nanoparticles (zinc and copper) that damaged the surface of the bacteria; zeolite further enhanced this property that killed the bacteria. Zeolite also acted as the stabilizing agent, preventing the agglomeration of the copper and zinc ions [6]. Copper oxide nanocomposites synthesized from copper oxide and zinc oxide nanoparticles with graphene oxide produced higher zones of inhibition against *Staphylococcus aureus.* Copper oxide nanocomposites (produced by cuprous oxide on the graphene nanocomposites sheets) are also known to produce bactericidal effects by producing reactive oxygen species such as hydrogen peroxide and super oxide anions. Hydrogen peroxide has a 70% bactericidal effect on Gram-negative bacteria (*Escherichia coli*) and a 65% bactericidal effect on Gram-positive bacteria (*Staphylococcus aureus)*, respectively. 

### 2.3. Antibacterial Mechanism of Action of Zinc Nanocomposites 

Ghosh et al. [7], in their study, reported that the mechanism by which the synthesized silver zinc oxide nanocomposite exhibited antibacterial activity may be due to the binding of nanocomposites to the cell wall of the bacteria; they were unable to predict the exact mechanism. To understand the mechanism of the zinc oxide nanoparticles coupled with graphene oxide nanosheets nanocomposite, Prema et al. [8] carried out analysis for DNA fragmentation, lactate dehydrogenase leakage, and ROS analysis. Prema and her colleagues reported that the chromosomal DNA was the same, but there was increase in lactate dehydrogenase leakage and reactive oxygen species in all the microbes upon exposure to zinc oxide nanocomposites. Thambidurai et al. [9] attributed the antibacterial activity of nickel and zinc oxide nanocomposites to their large surface area and increase in the production of the ROS that killed the bacteria.

### 2.4. Antibacterial Mechanism of Action of Gold Nanocomposites 

Huang et al. [10] synthesized gold nanoparticles using a photothermal agent under the near infrared (NIR), and it had a bactericidal effect on the pathogenic bacteria. They synthesized polygonal shaped nanoparticles and appended them with vancomycin. These nanocomposites of gold vancomycin binded to the peptide part D-Ala-D-Ala in the pathogenic bacteria cell and caused the transfer of heat from the light source into the cell, causing its bactericidal effect; this killed more than 99% of the pathogenic bacteria, both Gram-positive and Gram-negative, and antibiotic-resistant bacteria [11]. 

In another study, Perni and his research team synthesized light-induced polymer nanocomposites made up of methylene blue and gold nanoparticles [11]. These nanocomposites’ gold nanoparticles exhibited bactericidal activity against methicillin-resistant Gram-negative bacteria *Escherichia coli* and Gram-positive bacteria *Staphylococcus aureus*. Attributed to the release of singlet oxygen and ROS from methylene blue due to interaction of light on the surface of the polymer, the presence of the gold nanoparticles enhanced the release of reactive oxygen species, further enhancing the bactericidal property. This may be of benefit in biomedical applications. Nirmala et al. [12] reported the bactericidal activity of gold nanoparticles coated with hydroxyapatite immaculate; they reported that the hydroxyapatite in its unaltered form had no harmful effect on the bacterial, but together with metal nanoparticles it had the bactericidal properties. The bactericidal effect was due to the production of electrons or ROS that bind to the membrane or surface of the plasma membrane and penetrate inside, causing the bactericidal effect. These nanocomposites have large surface area volume and are hyperbranched, which offers electrons to acts as a best antibacterial agent, but these nanoparticles are known to cause toxicity to osteoblast cells in the tissue. Hence, their use may be limited due to this factor. 

In another study, the nanocomposites synthesized from gold nanoparticles with poly-thiophene exhibited a significant bactericidal effect. The bactericidal properties were attributed to their ability to penetrate the cell membrane and kill the bacteria. This may be an excellent choice in preventing biofilm formation and inhibiting the gastrointestinal pathogens. Gold poly-thiophene nanocomposites have significant potency in eliminating the pathogen and are not toxic to human cells. Hence, this nanocomposite may be an excellent choice for therapeutic purposes for humans.

Polymer-based gold nanocomposites have shown bactericidal activity. Regiel-Futyra et al. (2015) synthesized polymer-based gold nanocomposites; they used chitosan flakes and gold chloride solution, and chitosan-based nanocomposites exhibited bactericidal activity against *Staphylococcus aureus* (ATTC 25923) and *Pseudomonas aeruginosa* (ATTC 27853) [13]. The polymer puffed up to reach the cell membrane of the bacteria, causing damage to the cell. A similar type of work was reported by Mendoza et al. [14]; they synthesized chitosan-based gold nanocomposites with two different gold solutions. These nanocomposites exhibited significant bactericidal effects without causing any damage to the human cell.

When antimicrobial peptide daptomycin was combined with gold nanoclusters to form hybrid covalent conjugates of nanocomposites, these hybrid nanocomposites were able to produce pores or openings in the membrane through which they entered and damaged the DNA, killing the bacterial cell. The mechanism by which nanocomposites prepared by conjugating iron oxide with gold nanoparticles exhibited the antibacterial activity was attributed to the attraction between positively charged ions (iron and gold) with the negatively charged ions on the bacterial surface, causing them to stick to the surface and resulting in death of the bacteria.

Metal and metal-based NPs exhibit antimicrobial properties; they exhibit antibacterial antiviral properties. The antiviral or the virucidal properties of some of the nanoparticles and nanocomposites are discussed briefly in the following paragraph. 

In the Guangdong province of China, in 2003, COVID-19 caused severe respiratory syndrome, affecting 8000 people in 37 countries with a low mortality of 10%. In 2012, severe respiratory syndrome MERS (Middle East respiratory syndrome) [15,16] affected individuals on the Arabian Peninsula with a high mortality rate (40%). In December 2019, in Hubei province, China, COVID-19 first appeared and spread across the world rapidly. Later, WHO declared it a pandemic [17,18]. Among nanoparticles, metal and metal oxide nanoparticles based on gold, silver, copper, and zinc are known to exhibit extensive antimicrobial properties, including viruses, bacteria, and fungi [19]. Silver nanoparticles are known to cause destruction to viruses [20,21], metal oxides, copper, and iron [22]. When silver and gold nanoparticles are associated with polymers and textiles, they confer viricidal properties to them [23]. Graphene oxide (GO) showed potent antiviral activity with nonionic polymer polyvinylpyrrolidone (PVP), but with cationic polymer poly-diallyl-dimethylammonium (PDDA) it did not show antiviral activity. GO also caused destruction of virus before the entry, further emphasizing the antiviral activity [24]. These are some of the methods that directly act on the virus inside the host. Another way to control the viral infection is to disinfect the surfaces that act as sources of contamination due to discharge from affected persons. The development of antiviral surfaces is a new concept that is currently picking up owing to the rise in viral infection. Lishchynskyi et al. (2022) extensively reviewed articles, highlighting the classification of modern antiviral surfaces, and the impacts of nonspecific and specific interaction on virus deposition. They also outlined the synthesis and characterization of nanostructured antiviral surfaces and lastly on the effects of different antiviral surfaces on different viruses [25].

## 3. Types of Metal–Polymer Nanocomposites with Antibacterial Properties Based on Metals (Silver, Copper, Zinc, and Gold)

### 3.1. Silver-Based Nanocomposites

Silver nanoparticles have been widely used as an antibacterial agent in metal–polymer nanocomposites [26]. Silver has a broad-spectrum antibacterial effect and can disrupt the bacterial cell membrane [27,28], leading to cell death [29]. Silver-based nanocomposites have been used in medical applications [29] such as wound dressings [30], orthopedic implants [31], and catheters [32]. The use of metal nanoparticles, specifically silver nanoparticles (AgNPs), as antimicrobial agents in medicine is a crucial application. When AgNPs are dissolved in an aqueous solution, Ag+ ions interact with microorganisms and have various antibacterial mechanisms. One of these mechanisms involves Ag^+^ ions binding to sulfur and phosphorus groups in the proteins of the cell wall and plasma membrane of bacteria, causing protein dysfunction and threatening the organism’s life [33,34]. Ag^+^ ions can also create holes in the microorganism’s membrane by binding to negatively charged parts, leading to the flow of cytoplasmic contents out of the cell, dissipating the proton gradient across the membrane, and ultimately causing cell death [35,36]. The biohazards of silver nanoparticles are well documented [37]. Ag^+^ ions inside the cell can disturb the function of the bacteria’s electron transport chain, and they can interact with bacterial DNA and RNA, inhibiting cell division [38,39].

Several factors affect the activity of AgNPs, including size, shape, and coating, as well as the medium’s parameters such as the presence of light, oxidative species, other potential ligands for silver, and ionic strength. These factors can impact various phenomena that contribute to the increase or decrease of the antibacterial activity of AgNPs through complex pathways. Table 1 shows the biomedical applications of Ag–polymer nanocomposites and their sources and properties.

### 3.2. Copper-Based Nanocomposites

Copper has also been used as an antibacterial agent in metal–polymer nanocomposites [61]. Copper ions can disrupt the bacterial cell wall and inhibit bacterial growth by inducing cell membrane [62] or intracellular protein and DNA disruption [63] and generation of reactive oxide species [64].

The incorporation of antimicrobial metals into a polymer can result in the creation of composite materials, but the effectiveness of this method is dependent on both the intended use of the composite and the type of polymer used. The metal can be added to the surface of the polymer or embedded within the matrix [65]. The use of polymers in creating nanocomposites with antimicrobial properties is not limited to providing support for nanoparticles as the polymer itself can enhance the antibacterial performance of the composite. This is due to the synergy between the polymer and the nanoparticles, the long-term ion release ability of the polymer, and the increased surface area resulting from the fine dispersion of nanoparticles within the polymer [65]. There are two general approaches for preparing polymer/metal nanocomposites, based on where the nanoparticles are produced. The first is in situ, which involves using the polymer matrix as the reaction medium. The second is ex situ, in which the particles are synthesized separately and then added to the polymer with a dispersion medium [65]. Table 2 illustrates the several synthetic and natural sources of copper–polymer nanocomposites.

### 3.3. Zinc-Based Nanocomposites

Zinc has also shown antibacterial properties and has been used in metal–polymer nanocomposites. Zinc ions can interfere with bacterial metabolism and inhibit bacterial growth. Zinc-based nanocomposites have been used in medical applications such as dental materials and wound dressings (Table 3).

The misuse of antimicrobial agents has led to the emergence of multidrug-resistant bacterial strains at an alarming rate, resulting in an increase in infectious diseases and associated mortality [103]. This resistance can arise through mutations, horizontal gene transfer, or adaptive changes [104]. The chronic and intense use of antifungal agents also leads to decreased sensitivity and the production of resistant strains, along with severe toxic effects. Biofilm formation by microbes also shields them from the action of antibiotics [105], making it crucial to investigate novel antimicrobial agents that can bypass multidrug resistance and biofilm-mediated protection [106]. Inorganic metal oxides, such as ZnO, have shown promise as antibacterial agents by damaging the cell membrane and inducing oxidative stress [107]. ZnONP, in particular, exhibits higher photocatalytic activity, increased biocompatibility, and selectivity, along with heat resistance and durability. Incorporating ZnONP into antimicrobial therapeutics could reduce health hazards associated with traditional antimicrobial agents [108]. The mechanism by which NPs exhibit photocatalytic activity is that when the NP absorbs a particular wavelength of light, it causes the excitation of the particles and causes the movement of electrons from the valence band to the conduction band, resulting in the pore formation and generation of free hydroxyl ions and radical anions, which may cause the death of bacteria [109].

**Table 3:** Zinc–polymer nanocomposites and their biomedical applications.

No.	Polymer Name	Source	Properties	Uses	Ref.
1	Chitosan hydrogel	Synthetic	30 nm	Antimicrobial	[110]
2	Cellulose	Synthetic	65 nm	Photocatalytic and antibacterial	[111]
3	Agar biopolymer	Synthetic	20 nm	Antibacterial and anticancer	[112]
4	Gelatin/tragacanth	Synthetic	10.6 nm	Antimicrobial biomaterials for food packaging	[113]
5	Chitosan	Natural	25 to 70 nm	Antimicrobial	[114]
6	Chitosan	Synthetic	20–150 nm	Antibacterial and photocatalytic	[115]
7	Low density polyethylene (LDPE)	Synthetic	~17 nm	Antimicrobial	[116]
8	Chitosan	Natural	24 nm	Antibacterial and photocatalytic	[117]
9	Alginate beads	Natural	20–100 nm	Antimicrobial agent for water disinfection	[118]
10	Polyvinyl alcohol	Synthetic	-	Antimicrobial coating	[119]
11	Polyaniline	Synthetic	61.6 nm	Antibacterial	[120]
12	Poly(3-hydroxybutyrate-co-3-hydroxyvalerate)	Synthetic	3.5, 25 nm	Food packaging and food contact surface applications	[121]
13	Multiwalled carbon nanotubes (MWCNTs)	Synthetic	-	Food packaging	[122]
14	Hydroxyethyl cellulose, carboxymethyl chitosan (CMCS) composite/film	Synthetic	-	Antibacterial, food packaging	[123]
15	Graphene oxide, composite resins	Synthetic	-	Antimicrobial	[124]
16	Mahua-oil-based polyurethane/chitosan/nano ZnO composite	Synthetic	30 nm	Food packaging	[125]
17	Polyvinyl (alcohol)/chitosan/nano zinc oxide hydrogels	Synthetic	30 nm	Wound healing	[126]
18	AZO-Np in Guar gum/polyvinyl alcohol composite fiber mats	Semisynthetic	Less than 50 nm	Antibacterial	[127]
19	Chitosan–alginate–gelatin and chitosan–bentonite–gelatin films with ZnO	Natural	Nanoscaled	Skin burn healing	[128]
20	Aminoalkylsilane-grafted bacterial nanocellulose	Synthetic	30 nm	Multifunctional wound dressing, antibacterial	[129]
21	PVA and xylan, nanoscaled ZnO	Synthetic	-	Bacteriostatic films	[130]
22	Oxidized sodium alginate and its electrospun bio-hybrids	Synthetic	-	Wound healing	[130]

### 3.4. Gold-Based Nanocomposites

Gold nanoparticles have been studied for their antibacterial properties. Gold nanoparticles can damage bacterial DNA and inhibit bacterial growth. Gold-based nanocomposites have potential applications in medical devices and sensors.

Gold nanoparticles have emerged as a primary candidate in a multitude of fields, such as nanobiotechnology, tissue engineering, and drug delivery, due to their excellent properties. Their strong affinity with organic species and high electrical conductivity make them suitable for applications in chemical sensing and drug delivery technologies.

In therapeutic, medical, and pharmaceutical fields, gold nanoparticles are used for a variety of purposes. Gold nanorods have proven to be effective in photothermal tumor therapy [131], while biosensors have been developed using gold nanoparticles to detect *Bacillus anthrax* [132]. Gold nanostars are also used for cancer therapy [132,133] and biological labeling [134]. Nanobelt gold nanoparticles serve as transducers and resonators and can be used as nanoscale sensors in medical approaches [132,135]. Nanoshells have potential for photonic crystals, fluorescent diagnostics, improving photoluminescent labels, catalysis, avoiding photodegradation, research in bioconjugates, and chemical and colloidal studies [136]. Additionally, gold nanoparticles are now widely used as antibacterial agents against a broad range of microorganisms [137] due to their biocompatibility, low cost, simple production, and high impact. Gold nanoparticle nanocomposites offer several advantages [138,139]. As shown in Table 4, gold–polymer nanocomposites are used as antimicrobials and for wound healing.

## 4. Synthesis of Metal–Polymeric Nanocomposites

**Figure 3:**
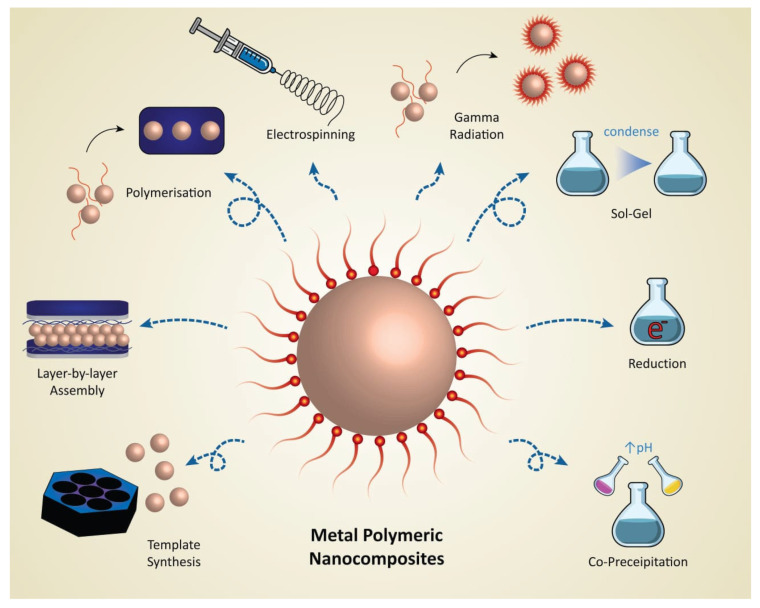
Different synthetic approaches to produce metal–polymer nanocomposites.

Various approaches produce functional polymer composites containing metal nanoparticles (Figure 3), including reduction of a metal salt [154], ball milling [155], plasma polymerization [156], co-evaporation [157], and co-sputtering [158]. The goal is to achieve good control of the metal filling factor, filling factor profile, and particle composition [16,17,18,19,20,21,22,23,24].

### 4.1. In Situ Polymerization

**Figure 4:**
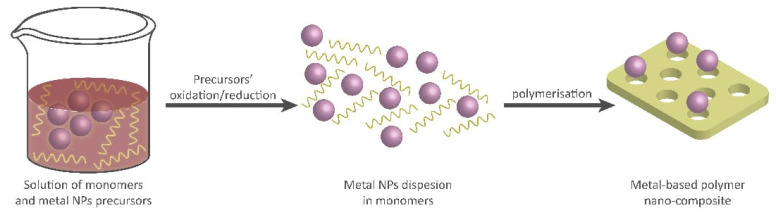
In situ polymerization techniques for the synthesis of nanocomposites.

The in-situ polymerization method involves the simultaneous polymerization of monomers and the reduction of metal ions to form copper nanoparticles within the polymer matrix (Figure 4). One common in situ polymerization methods is emulsion polymerization [159]. This method involves the dispersion of a hydrophobic monomer in water using surfactants. Metal salts are then added to the emulsion, followed by the addition of a reducing agent to initiate polymerization and nanoparticles formation [160,161]. 

### 4.2. Chemical Reduction

The reduction of chemically bound atoms in nonpolar media or metal cations in polar media (usually aqueous) is the most commonly used chemical method for obtaining metal–polymer nanocomposites [162]. This method offers several benefits such as speed, simplicity, and cost-effectiveness and enables the production of NPs with well-controlled sizes and shapes, facilitated by reducing and stabilizing agents that prevent NP agglomeration. The size and distribution of particles are influenced by factors such as redox potential, temperature, reagent and additive concentrations, and solvent type. It is worth noting that the in situ synthesis of MPNs also relies on the molecular weight, concentration, and chemical properties of the polymer. Additionally, the hydrophilicity, hydrophobicity, or amphiphilicity of the polymers, along with their subsequent configuration in a solvent, also have a crucial impact [163].

#### 4.2.1. Engineering Polymers

Several studies have explored the use of various polymers to immobilize silver nanoparticles for their antimicrobial properties. One study described the coating of PET fibers with sericin-capped silver nanoparticles, where the fibers were first modified with a silane coupling agent and then coated using a dip-coating method [164]. Another study discussed the synthesis of chitosan-silk fibroin nanofibers containing silver nanoparticles for use as an antibacterial wound dressing, where the AgNPs were incorporated via electrospinning along with curcumin for sustained release [165].

#### 4.2.2. Carbohydrates and Biopolymers

AgNPs can be combined with polymers using lignin as a stabilizer, which is a natural polymer found in plants. Lignin can be modified to make it compatible with other materials. In a study highlighted by the authors, lignin was used to stabilize AgNPs, resulting in a material with good antibacterial activity against *E. coli* [166]. The addition of organo-clay improved the interfacial adhesion between the AgNPs and the polymer matrix, resulting in a better dispersion and improved mechanical properties of the nanocomposite films. Thymol, which was also dispersed in the polymer matrix, improved the thermal stability and antioxidant properties of the films [167].

#### 4.2.3. Dendrimers as Templates and Hosts for Metal Nanoparticles

Two studies described different methods for synthesizing hybrid nanoparticles. One study used a DNA dendrimer template to control the size and shape of copper nanoparticles [168], while the other study described the grafting of a PPI dendrimer onto gold nanoparticles using a multistep process. Both methods involved the use of functional groups for subsequent modifications [169].

### 4.3. Electrospinning

Electrospinning is a popular method for synthesizing metal–polymer nanocomposites, where an electric field is applied to a polymer solution or melt, resulting in the formation of nanofibers with diameters in the nanometer range. The incorporation of metal nanoparticles or nanowires into the polymer matrix during the electrospinning process enables the creation of nanocomposites with uniform metal distribution. These materials offer improved mechanical, electrical, and optical properties, making them attractive for various applications, including sensors, catalysis, and energy storage. One paper described a synthesis method for producing flexible nylon–ZnO core–shell nanofiber mats with enhanced photocatalytic activity. The method involves electrospinning to produce the nylon core fibers, followed by atomic layer deposition (ALD) to coat the ZnO shell onto the nylon fibers. The resulting nanofiber mats exhibit high flexibility and mechanical strength due to the presence of the nylon core, and the ZnO shell provides photocatalytic activity for potential environmental applications [170].

Another method involved dissolving PVP in a solvent system, generating gold nanoparticles by laser ablation in a PVP solution, and then electrospinning the resulting PVP/Au NP solution. The nanofibers were deposited on a cylindrical metal collector, and the electrospinning was carried out under controlled conditions. For comparison, electrospinning of PVP in the solvent system without laser ablation was also performed [171]. 

### 4.4. Layer-by-Layer Assembly

The layer-by-layer (LbL) assembly technique is a versatile and powerful method used to synthesize polymer nanocomposites by sequentially depositing oppositely charged species [172]. The technique has been used to fabricate various nanocomposites with tunable compositions and structures, such as a humidity sensor Co_3_O_4_/PSS [173], a bio-interface GO/PAH with excellent mechanical properties [174], a gas sensor based on polypyrrole (PPy)/TiO_2_ [175], a supercapacitor halloysite/polyaniline, and a foam coating nanoclay/PEI with customizable properties. The LbL assembly technique is capable of achieving nanoscale precision over the thickness and high content of well-dispersed nanomaterials. By incorporating nanomaterials into the polymer matrix, the physicochemical properties of polymer composites can be modified or altered [176].

In the field of LbL assembly, nanoparticles have evolved from being simple additives to become key components that drive the development of various applications, including smart coatings and drug delivery systems. Despite some disadvantages, the advantages of using nanoparticles in LbL assembly outweigh them, and future trends suggest that they are indispensable for achieving advanced coatings with controllable properties and responsiveness to various stimuli [177].

In a recent study, LbL assembly was employed to generate nanoscale antioxidant coatings using cerium oxide nanoparticles (CONPs) and alginate on various materials. The uniformity and scale of the LbL assembly were characterized using ellipsometry, which demonstrated predictable multilayer formation. The versatility of CONP coatings on three-dimensional surfaces was explored by coating silica and alginate microbeads with increasing numbers of alternating layers of CONP/alginate. Confocal microscopy images showed ultrathin coatings formed onto the different spherical materials, with increasing fluorescence intensity correlating with increasing layer number. The resulting nanoscale antioxidant coatings were able to protect encapsulated cells from reactive oxide species (ROS)-mediated damage while preserving capsule permeability and cellular function. This approach provides retention of catalytic activity, control of localization, and mitigation of cytotoxicity, making it a promising strategy for a variety of applications in tissue engineering and drug delivery [178].

Another study described a spray-assisted method for the alignment of silver nanowires in LbL assembled films. The method involves the use of a spray gun to deposit the LbL films onto a substrate while simultaneously applying a magnetic field to align the silver nanowires. The authors showed that this approach could result in highly anisotropic nanocomposite films with controlled thickness and composition [179]. 

**Figure 5:**
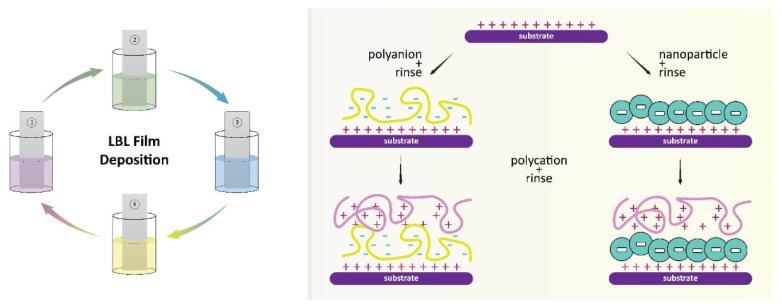
Layer-by-layer film deposition method for the synthesis of nanocomposite with inorganic species.

Figure 5 illustrates the process of layer-by-layer assembly mechanisms, which demonstrate the alternate deposition of positively and negatively charged components onto a substrate. The LBL assembly allows for the introduction of various nanospecies, such as inorganic nanoparticles (NPs) and polymers, at the same time with high loading in the assembled layers. The assembly of each layer can be easily modulated to achieve the optimal structure for a specific application, and the prepared layers can be assembled with controlled variable thicknesses for generating biocompatible coatings. The ability of LBL to control the coating thickness, properties of the nanocomponents, and economic use of raw materials make the assembly tool greatly superior to other methods. This approach provides the opportunity to combine the electronic, optical, and magnetic properties of inorganic nanostructures with unique physical responses. The resulting LBL-based thin films have been applied to various applications, including drug delivery systems and thin membranes.

### 4.5. Template Synthesis

Template synthesis of metal–polymer nanocomposites is a powerful approach that allows the production of highly ordered nanoscale hybrid materials with unique properties. The method involves the use of a template, usually a self-assembled monolayer or a block copolymer, to direct the formation of the metal nanoparticles within the polymer matrix.

**Figure 6:**
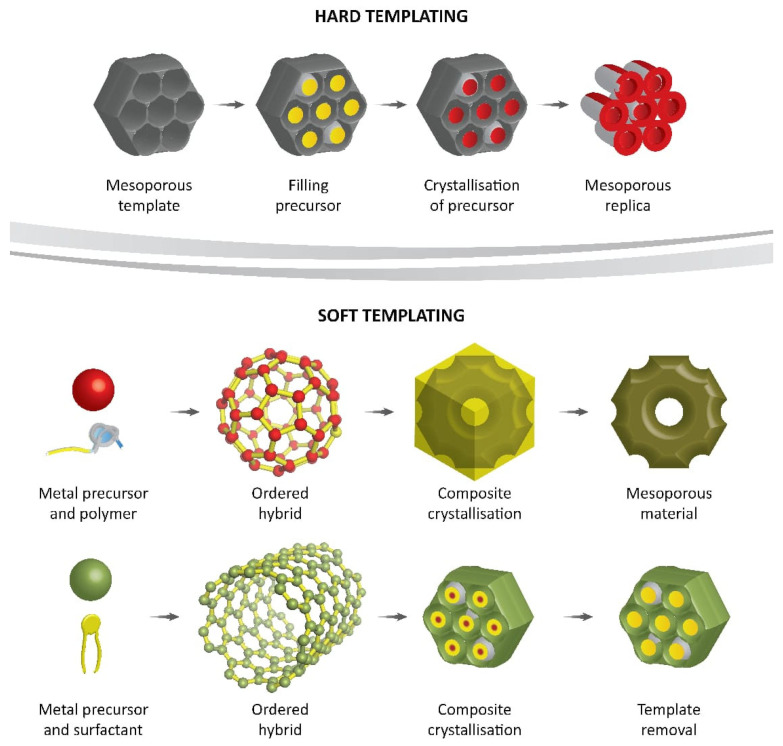
Hard template and soft approach for the synthesis of nanoscale architecture in the preparation of various polymeric nanocomposites containing inorganic nanoparticles.

The template provides a highly ordered nanoscale architecture, which enables the control of the size, shape, and distribution of the metal nanoparticles within the polymer matrix. This control over the metal nanoparticle morphology leads to improved mechanical, electrical, and optical properties, making them useful in a wide range of applications [180]. There exist two primary categories of template-based synthesis methods, namely, the hard template approach, which employs a physical mold for the growth of nanostructures within composite polymers [181], and the soft template approach, which utilizes the self-assembly of composite polymers [182] (Figure 6).

#### 4.5.1. Hard Template Approach 

The hard template technique is a versatile and effective method for synthesizing metal–polymer nanocomposites. This method involves using a physical template, such as colloidal nanoparticles or nanosized channels, to control the shape and size of the resulting nanocomposites. The hard template technique can involve both chemical and electrochemical polymerization processes, depending on the specific reaction parameters. Colloidal nanoparticles have been commonly used as templates for synthesizing 0D metal–polymer nanocomposites, with the resulting core–shell structures highly dependent on the size of the nanoparticles. This method offers advantages such as ease of synthesis, even size dispersion, and large quantity availability [183].

#### 4.5.2. Soft Template Approach 

Soft template synthesis is a technique used to fabricate conducting polymer nanocomposites (CPNCs) by confining CP polymerization within surfactant micelles. This method is cheaper and less intricate than hard template synthesis and can produce CPNCs in large quantities. The morphology and size of the CPNCs depend on parameters such as microstructure, morphology, and concentration. Cationic surfactants such as octyltrimethyl ammonium bromide (OTAB), decyltrimethyl ammonium bromide (DTAB), and cetyltrimethyl ammonium bromide (CTAB) are used in micelle formation [184]. Polypyrrole (PPy) nanostructures of different shapes can be produced by varying the concentration of monomer and surfactant. Recently, PPy-based materials have been used to fabricate organic polymeric-oriented thermoelectric hybrid materials and composites, as well as supercapattery attributes of CeO_2_ nanoparticles. The inclusion of Cu–Al_2_O_3_ nanoparticles in PPy/Cu–Al_2_O_3_ nanocomposites enhances their electrical conductivity and gas sensing abilities [185].

### 4.6. Coprecipitation

The coprecipitation method involves obtaining a uniform mixture of two or more cations in a solution during the precipitation reaction. This method is widely used for synthesizing composites that contain multiple metal elements. The coprecipitation reaction consists of several processes, including nucleation, growth, coarsening, and agglomeration [186]. It is a significant technique for synthesizing composites containing multiple metal elements in a homogeneous solution during precipitation. This method is preferred over solid and vapor phase techniques due to its advantageous properties, such as short diffusion paths, high product purity, less agglomeration, simplicity, uniform and controllable particle size, and cost-effectiveness [9,116,187,188,189]. One recent example of this method is the fabrication of mesoporous iron–manganese bimetal oxide nanocomposites using the aeration coprecipitation technique. The method involves separately preparing a molar ratio of 2:1 of manganese sulfate and ferric chloride, mixing them at 70 °C with stirring at 50 rpm, adding 10% NH_4_OH to obtain a pH of 9, and keeping it at 50 rpm for 4 h to obtain the precipitated nanocomposites [190]. Similarly, a NiFeO_4_ nanocomposite was synthesized using the coprecipitation method, where a 1:1 molar ratio of FeSO_4_.7H_2_O and NiSO_4_.7H_2_O was used at a pH of 12, and hydrazine hydrate acted as the reducing agent [191].

### 4.7. Sol–Gel Process 

The sol–gel method is a wet-chemical technique that employs a chemical solution or colloidal particles to create a cohesive network or gel. It uses metal alkoxides or metal chlorides as precursor materials. This process is also referred to as chemical solution deposition, which comprises various steps such as hydrolysis, polycondensation, gelation, aging, drying, densification, and crystallization. The sol–gel process is advantageous for producing nanocrystalline powders at relatively lower temperatures and pressures [186]. The size, morphology, dimensions, composition, and structure of nanomaterials play a crucial role in their various applications such as catalysis, sensing, and antibacterial. The sol–gel process is an important technique for the synthesis of nanomaterials due to its ease of operation, low-temperature reaction conditions, and ability to produce products with defined size, high purity, and homogeneity [192,193,194,195]. Ternary CuO/TiO_2_/ZnO nanocomposites were successfully synthesized using the sol–gel method. This transition metal oxide-based nanocomposite was found to enhance the photocatalytic degradation of methylene blue (MB), an organic dye [196]. In another study, a NiO/TiO_2_ nanocomposite was also prepared using the sol–gel method and was used for the degradation of MB organic dye [197]. Similarly, a ZnO/CuO nanomaterial was prepared using the sol–gel technique, where calculated amounts of zinc nitrate and copper oxide were dissolved and dispersed in the solvent, followed by the addition of PVA and stirring in an ultrasound bath at 353 K to obtain a homogeneous gel. The gel was then dried at 773 K for 8 h to obtain the required nanocomposite [198]. In yet another study, a TiO_4_–Ag nanocomposite was reported to exhibit antibacterial activity against *E. coli* and was also synthesized using the sol–gel route of synthesis [199].

### 4.8. Gamma Radiation

**Figure 7:**
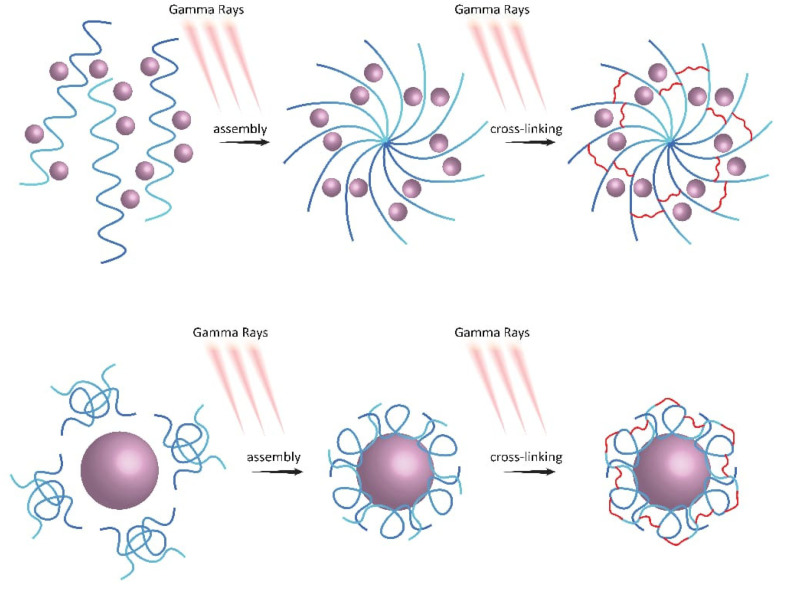
Gamma-rays-catalyzed synthesis of nanoparticles and crosslinked polymers, nanocomposite with nanoparticles.

Gamma irradiation has become a highly effective method for synthesizing metal–polymer composites by creating random reducing and oxidizing agents with significant redox potentials in aqueous solutions [200], as depicted in Figure 3 and Figure 7. To enhance the concentration of these agents, additives such as alcohols or NO_2_ can be employed [201], initiating chemical reactions involving metal ions, metal complexes, monomers, and natural or synthetic polymers [202]. Gamma-irradiation-based synthesis of NPs in water generates highly reactive initiators for forming both inorganic and organic NPs. However, this approach is challenging because the reaction pathways are not always well established and can be quite complex. Two primary methods of synthesizing nanocomposites with gamma rays have been identified: one-step and two-step. One-step synthesis generates complete nanocomposites by combining a monomer or polymer with metallic ions and exposing them to gamma radiation in an inert atmosphere, while two-step synthesis involves first creating nuclei, either metallic or polymeric, and then completing the shell.

In summary, gamma ray synthesis of nanocomposites offers a versatile and effective way to create new materials with unique properties and potential applications in various fields. The choice of metallic nucleus, polymeric shell, and synthesis methodology can influence the type and shape of the resulting nanoparticles.

## 5. Challenges of Synthetic Approaches

During the synthesis of nanoparticles (NPs), one of the main difficulties is the unwanted aggregation of particles, which can sometimes be irreversible even when using a continuous distribution medium. Therefore, achieving a homogeneous redispersion of NPs is not always easy and can even lead to degradation and loss of functionality. To preserve the properties and purity of NPs, it is highly advantageous to use approaches that do not require solvent extraction.

To address this challenge, several methods have been developed to synthesize NPs atom by atom in gas, solid, or liquid phases [203]. In the liquid phase, metallic NPs are chemically synthesized using traditional methods such as reducing precipitation and coprecipitation. For organic NPs, crosslinking of organic macromolecules and polymerization are commonly used, with environmentally friendly synthesis methods involving micro/nanoemulsions assisted by photochemical and electrochemical methodologies. These methods require precursors, reducing agents, particle capping agents, stabilizers, and solvents [204].

However, common chemical processes in the synthesis of nanomaterials can be complicated by the contamination of NPs with chemical precursors and additives during the synthetic procedures. This aspect is particularly important for applications of NPs in electronics or healthcare. As the complexity of synthesis increases, so too do the costs, toxicity, operational safety, and environmental risks. Additionally, there is little known about the toxicity of NPs due to the lack of specific toxicity data.

## 6. Characterization of Metal–Polymeric Nanocomposites

Proper characterization of metallic and polymeric nanoparticles is crucial for understanding their characteristics, particularly in terms of their particle size, distribution, and shape [205]. Various techniques are typically employed for nanoparticles characterization, including transmission electron microscopy (TEM), atomic force microscopy (AFM), and scanning tunneling microscopic (STM) imaging to determine size and surface morphology. Additionally, electron spin resonance spectroscopy (ESR), nuclear magnetic resonance spectroscopy (NMR), extended X-ray absorption fine structure (EXAFS), X-ray, energy dispersive spectroscopy (EDS), and energy-dispersive X-ray spectroscopy (EDAX) are commonly used for determining structure and composition. Other methods such as UV/vis and infrared (IR) spectroscopy provide valuable information about the plasmonic surface as well as chemical groups and bonding on the particle. Furthermore, gel permeation chromatography (GPC) in combination with a static light scattering (SLS) detector is used to determine the average molecular weight of polymeric NPs. Overall, appropriate characterization methods are essential for gaining a comprehensive understanding of metallic and polymeric nanoparticles.

## 7. Applications and Limitations

The biomedical field is using nanotechnology to prevent microbial infection and the disease caused by the infection. This is carried out by using the atomic size of the active material. For example, AgNPs have strong antibacterial properties [206]. Nevertheless, they also have cytotoxic properties. This can be reduced by implanting or inserting the, with suitable polymer grids [207]. It (the presence of Ag in polymers) has also improved L929 cell attachment and development [208]. The antibacterial properties are due to the release of silver ions from the polymer nanocomposite [209]. Similarly, coating in light of polymer/Cu nanocomposites was due to the discharge of metal ions that exhibited significant, or prevented the development of, fungi and pathogenic microorganisms [210]. Controlled discharge of silver ions from metal–polymer nanocomposites can be used in various biomedical applications or fields, such as wound dressings, orthopedic implants [31], and catheters [32], paint or coating biomedical facilities or doctor’s facilities, households, or aviation. The antibacterial efficiency of the silver polymer nanocomposites on Gram-positive and Gram-negative bacteria was summarized above in this article. The other applications of polymer-based silver nanocomposites may be in the prevention of microbial infection in the food industries, in decontamination of water, in wound dressing, or inhibiting the growth of bacteria in injuries. Temperature-responsive nanohybrids synthesized form AgNO_3_ may be used in the preservation of solid substrates, as photothermal agents against microorganisms and against tumors, and in the development of surgically modified face masks [211]. Research using LDPE/Cu nanocomposite as intrauterine material has been explored [212]. Studies have demonstrated its effectiveness, and it may be utilized in place of conventional material. Gold-based nanocomposites have potential in medical devices, sensors, tissue engineering, and drug delivery. Gold nanorods have proven effective in photothermal tumor therapy [131], while biosensors have been developed using gold nanoparticles to detect Bacillus anthrax [132]. Gold nanostars are also used for cancer therapy [132,133] and biological labeling [134]. Nanobelt gold nanoparticles serve as transducers and resonators and can be used as nanoscale sensors in medical approaches [132,135].

The antibacterial property of the metal/polymer nanocomposites may be challenged by some of the properties of the nanoparticles, such as their ability to aggregate together due to high surface free energy; they may oxidize and pollute air, and these properties may diminish the antibacterial properties of the metal–polymer-based nanocomposites. One of the areas of concern for the nanocomposites is the toxicological aspects, and many studies are being carried out in this respect. This may hinder its biomedical application. Hence, in vitro studies are required as in vivo studies may be difficult to apprehend. There are no reports yet on the unfriendly behavior of the nanoparticles, owing to their difference in physical properties such as the shape, size, chemical nature (hydrophobicity, etc.), chemistry, and the method that has been used in the synthesis, the target organs, or the cells. Similarly, there are difficulties with the in vivo test. Hence, standard tests are required to assess the exact fate of these nanoparticles in vivo. The nanoparticles may not be targeted and recognized by the body’s immune system due to its size and may cause toxic response [213]. It seems that there is no standard test to evaluate the bioconsistent reaction to nanoparticles. Other things that must be taken into consideration are that nanoparticles are smaller than the chemical compounds, the study period must be longer, and alteration in the cytotoxicity test is needed. The physical and chemical parameters are also to be considered, such as size, shape, and the surface. These drawbacks are to be considered and assessed in all research; similarly, the toxicity posed by the polymer needs to be considered and assessed in the future to keep the threats below the recommended limits. 

Future strategies for the development of more enhanced antibacterial nanocomposites depend on several factors. First, the use of a suitable polymer with therapeutic potential against multidrug-resistant microorganisms, which may be an alternate to the microbes resistant to antibiotics and against various microbes (fungi, viruses, etc.); secondly, to use green synthesis, plant-based polymers with antibacterial properties, and with fewer side effects, a new bactericidal nanocomposite may be developed. The other approach would include the use of smart polymer nanocomposites or stimuli-responsive polymer nanocomposites, which are those that contain one chemical and one physical property controlled in response to the stimulus. The stimulus may be external or internal. The external stimuli are concerned with temperature, light, electric current, and magnetic field strength. The internal stimuli are concerned with pH biological recognition, solvent type, and chemical recognition. Thus, by utilizing various properties of NPs, selective release of drug to the target can be achieved in the incorporation of external stimuli such as light pH, and sound; this may also increase the antibacterial efficacy and reduce side effects [214]. 

For any nanocomposites to be used in clinical trials, they must be studied extensively for their cytotoxic effects using effective in vivo studies. It is important to choose a suitable method for the synthesis of nanocomposites to be developed on a large scale for commercial purposes in order to substitute it for present existing antibacterial or antimicrobial agent. The advantages of the methods used in the synthesis are discussed above. More research investments are needed in this area to develop a potent antibacterial. 

## 8. Conclusions

The metal-based and metal–polymer-based nanocomposites show significant antibacterial potential and have various biomedical applications. These metal-based nanocomposites have shown significant antibacterial properties; however, some of the metal-oxide-based nanocomposites are toxic to human cells, whereas most of them show negligible toxicity. As mentioned above, the goal is to develop good control of metal filling factor, filling factor profile, and particle composition. There are various approaches in developing a desired metal–polymer nanocomposite. The chemical-reduction method offers various benefits in synthesis such as speed, simple technique, and cost-effectiveness with well-controlled shapes and sizes of the NPs. However, there are several factors that must be kept in mind, to name a few: temperature, redox reaction, nature of the polymer, etc., which can be addressed. Techniques such as electrospin can be used to create nanofibers, which can be loaded with antibacterial activity and can be used as wound dressings to cure injuries. In the layer-by-layer technique, nanoscale precision is achieved with high content of well-dispersed nanomaterials, and the polymer can be modified by altering its physicochemical properties. It can be used in the coating of the drugs; despite its limitations, the benefits the LBL offers outweigh the limitations, which may explain the choice to develop nanocomposites using this technique. Template techniques enable one to develop well-structured nanocomposites with desired shape and size of nanoparticles, and their well-organized distribution in the polymer matrix of this method allows the coprecipitation that contains a mixture of one or more elements. The product is pure with the least contamination and desired shapes and sizes; with few agglomerations, this method is simple and less costly. Methods such as the gamma ray method offer an effective way to develop versatile nanocomposites with various properties that can be employed for various applications. The metal-based and metal–polymer-based nanocomposites have shown various benefits in biomedical applications and as potential antibacterials. Further extensive scientific research is required to test their toxicity and carry out the in vivo study, so that they can be used for clinical trials and can be marketed as therapeutically enhanced antibacterials.

## Figures and Tables

**Table 1:** Silver–polymer nanocomposites and their biomedical applications.

No.	Polymer Name	Source	Properties	Uses	Ref.
1	Polyethylene glycol PEG	Synthetic	5 to 20 nm	Antimicrobial	[40]
2	Chitosan	Synthetic	20.3 ± 0.7 nm and 44.6 ± 0.3 nm	Antifungal	[41]
3	Modified polypropylene	Synthetic	26 and 41	Antibacterial	[42]
4	Poly-acrylonitrile (PAN)	Synthetic	30–95 nm	Antimicrobial activity, electrical conductivity, ultraviolet blocking, and catalytic activity	[43]
5	Poly (vinyl alcohol) (PVA)	Synthetic	40.49–44.77	Antimicrobial	[44]
6	Cellulose nanowhiskers	Cotton fibers	6 nm and 18 nm	Antibacterial	[45]
7	Chitosan hydrogels	Synthetic	4.45–9.22 nm	Antibacterial	[46]
8	Chitosan	Synthetic	190 nm	Antioxidant, antibacterial, hemolytic, and cutaneous wound healing.	[47]
9	Gelatin chitosan	Synthetic	3–6 nm	Antimicrobial and wound healing	[48]
10	Chitosan	Synthetic	35 nm	Antibacterial and wound healing	[49]
11	Poly (methyl methacrylate) with chitosan	Synthetic	37.2 ± 16.4 nm	Antibacterial	[50]
12	Chitosan	Synthetic	5.0–9.9 nm	Dental barrier membrane applications	[51]
13	Tragacanth/hydroxy-propyl methyl cellulose/beeswax films	Natural	8–10 nm	Antimicrobial, edible films	[52]
14	Polyacrylonitrile nanofibers	Synthetic	7–20 nm (AgNP); 450–700 nm (nanofiber)	Antibacterial, antioxidant	[53]
15	Reduced graphene oxide	Synthetic	<10 nm	Antibacterial	[54]
16	Chitosan-based carbon nitride-polydopamine silver composite	Synthetic	-	Wound healing antibacterial	[55]
17	Hemicellulose, chitosan/chitin, and glutaraldehyde	Synthetic	-	Wound healing dressings	[56]
18	Polyurethane and cellulose acetate	Synthetic	-	Wound healing	[57]
19	Arabinoxylan-co-acrylic acid, nanohydroxyapatite (nHAp), graphene oxide	Synthetic	20–100 nm	Bone tissue engineering, antibacterial, biocompatible	[58]
20	Carboxymethyl chitosan-polyamideamine alginate composite	Synthetic	158.0 ± 2.3 nm	Antibacterial, wound healing	[59]
21	P4VP, P (4VP-co-OEGMA246), and POEGMA246	Synthetic	20–60 nm	Optical application	[60]

**Table 2:** Copper–polymer nanocomposites and their biomedical applications.

No.	Polymer Name	Source	Properties	Uses	Ref.
1	Chitosan	Synthetic	160 nm	Antimicrobial, photocatalyst	[66]
2	Chitosan: Pluronic F127	Synthetic	~8 ± 2 nm	Antimicrobial	[67]
3	Starch	Natural	200 nm	Antimicrobial, antioxidant, and anticancer	[68]
4	Polyacrylonitrile	Synthetic	100–200 nm long, 80 nm wide	Antibacterial	[69]
5	Poly(3-hydroxybutyrate-co-3-hydroxyvalerate) (PHBV)	Synthetic	182.65 nm	Antibacterial	[70]
6	Polyurethane	Synthetic	47.51 nm	Antibacterial	[71]
7	Polystyrene	Synthetic	18–25 nm	Antimicrobial	[72]
8	Bacterial cellulose	Natural	25 and 35 nm	Antimicrobial	[73]
9	Cotton fabrics modified with polycarboxylic acids	Synthetic		Antibacterial	[74]
10	Montmorillonite	Synthetic	15.29 nm	Antibacterial	[75]
11	Reduced graphene oxide	Synthetic	5–40 nm	Antibacterial	[76]
12	Chitosan	Natural	17 nm	Antimicrobial, sporicidal, and biofilm-inhibitory activity	[77]
13	Polyethylene oxide	Synthetic	12 ± 6 nm	Preservation system for fruit salads	[78]
14	Chitin	Synthetic	52.1 ± 20 nm	Anticancer	[79]
15	Chitosan	Synthetic	11 ± 6 nm	Antibacterial	[80]
16	Cellulose	Natural	21–30 nm and 31–40 nm	Antibacterial	[81]
17	Carboxymethyl starch	Synthetic	30–50 nm	Antimicrobial, wound healing	[82]
18	Chitosan	Natural	40–110	Antimicrobial	[83]
19	Nanochitosan	Natural	18 to 40 nm	Antimicrobial	[84]
20	Cellulose gum	Natural	7 to 12 nm	Antimicrobial against UTI	[85]
21	Cellulose acetate	Synthetic	143.2 to 157.1 nm,	Antimicrobial, cell viability	[86]
22	Hypromellose polymer	Synthetic	50–60 nm, nanofibers	Antibacterial	[87]
23	poly(vinyl alcohol)	Synthetic	300 nm	Antimicrobial-air microfilter	[88]
24	Bacterial cellulose	Natural	10–100 nm	Antibacterial	[89]
25	Hydroxypropyl methyl cellulose hydrogel	Synthetic	3–17 nm	Antibacterial	[90]
26	Chitosan-capped NP	Natural	5–9 nm	Microbial-resistant nanocomposites	[91]
27	Poly(diallyldimethylammonium chloride) (PDADMAC), Poly(sodium 4-styrenesulfonate)(PSS)	Synthetic	50 and 70 nm	Antimicrobial coating	[92]
28	3-(Trimethoxysilyl)propyl methacrylate, mesoporous silica gel	Synthetic	~10 nm	Bactericidal, wound healing	[93]
29	Poly(piperizinamide)/copper composite membrane	Synthetic	1 nm pore size	Mitigation of hexaconazole from water and combat microbial contamination	[94]
30	Graphene oxide–chitosan composite	Synthetic	2.4 to 257.6 nm	Antibacterial and cytotoxic activities	[95]
31	Chitosan nanocomposite	Natural	Polygonal 30–50 nm	Antibacterial and electrical properties	[96]
32	Polyvinyl alcohol	Synthetic	1–100 nm	Antibacterial	[97]
33	Chitosan	Bio-synthetic	20–100 nm	Antibacterial activity	[98]
34	Chitosan nanocomposite	Natural, nanocomposite using *Sida cordifolia* extract	~	Antibacterial, anticancer activity against on breast and lung cancer cell lines	[99]
35	Multifunctional chitosan–gallic-acid-based nanocomposite	Natural	30 nm	Antibacterial, wound dressing	[100]
36	Polyacryonitrile	Synthetic	Nanofibers, nanoparticles, 100 nm	Antibacterial against resistant strains, MRSA	[101]
37	Nanoderivatives-modified chitosan/hyaluronic acid	Synthetic	5–10nm	Wound healing	[102]

**Table 4:** Gold–polymer nanocomposites and their biomedical applications.

No.	Polymer name	Source	Properties	Uses	Ref.
1	Chitosan/aminopropylsilane	Synthetic	3.99 nm	Antimicrobial	[140]
2	Chitosan	Synthetic	5–100 nm	Antimicrobial and wound-healing activities	[141]
3	Imidazole-molecule-capped chitosan	Synthetic	12.16 nm, 10.87 nm, 9.56 nm, and 8.56 nm	Antimicrobial	[70]
4	Chitosan	Natural	16.9 ± 2.0, 25.0 ± 4.0 and 34.1 ± 5.9 nm	Antibacterial	[142]
5	Chitosan	Synthetic	14 nm	Antibacterial	[14]
6	Carboxymethyl cellulose	Synthetic	10–90 nm	Antimicrobial and anticancer	[143]
7	Heparin-polyvinyl alcohol	Synthetic	less than 100 nm	Wound healing	[144]
8	Highly reduced graphene oxide	Synthetic	3.27 ± 0.02 nm	Antiproliferative	[145]
9	Black phosphorus/gold nanocomposites	Synthetic	Nanoscale	Antibacterial activity due to photothermal effect, oxidative stress, and physical membrane damage	[146]
10	Chitosan-capped gold nanoparticles coupled with ampicillin	Natural	ellipsoidal particles, 50–100 nm	Antimicrobial activity	[147]
11	Chitosan, (gold)/perlite (Au/Perl) nanocomposites	Natural	Mesoporous/spherical shape, 13–15 nm	Antibacterial, wound healing against MRSA	[148]
12	Sodium alginate nanocomposite with gold nanoparticles/polyaniline boronic acid	Synthetic	15–20 nm	Antibacterial, biocompatible	[149]
13	Chitosan, imidazole-molecule-capped chitosan	Synthetic	8.5–12.5 nm	Antimicrobial activity	[150]
14	Dialyzed natural polymer, fibroin, (gold) nanocomposite	Natural	20–30 nm	Zika virus vector larvicidal activities	[151]
15	Polyoxoborate matrix/nanocomposite of gold nanoparticles	Synthetic	Nanoscaled	Antibacterial	[152]
16	Poly(*N*- isopropylacrylamide) (PNIPAM) brushes	Synthetic	Nanoscaled	Nanosensor	[153]

## Data Availability

Data available within the text.
